# Universal simulation of absorption effects for X-ray diffraction in reflection geometry

**DOI:** 10.1107/S2053273324003292

**Published:** 2024-06-07

**Authors:** Johannes Dallmann, Jonas Graetz, Rainer Hock

**Affiliations:** a Institute for Crystallography and Structural Physics, Friedrich-Alexander-Universität, Erlangen-Nürnberg, Germany; Swiss Light Source, Switzerland

**Keywords:** absorption corrections, microstructure, ray-tracing, surface roughness, powder diffraction

## Abstract

Analytical calculations of absorption corrections for X-ray powder diffraction experiments on non-ideal samples with surface roughness, porosity or absorption contrasts from multiple phases require complex mathematical models to represent their material distribution. In a computational approach to this problem, a practicable ray-tracing algorithm is formulated which is capable of simulating angle-dependent absorption corrections in reflection geometry for any given rasterized sample model. Single or multiphase systems with arbitrary surface roughness, porosity and spatial distribution of the phases in any combination can be modeled on a voxel grid by assigning respective values to each voxel. The absorption corrections are calculated by tracing the attenuation of X-rays along their individual paths via a modified shear-warp algorithm. The algorithm is presented in detail and the results of simulated absorption corrections on samples with various surface modulations are discussed in the context of published experimental results.

## Abstract 2

A ray-tracing algorithm for the simulation of absorption effects for arbitrary rasterized samples is presented. The algorithm is used to analyze how the absorption correction is influenced by the lateral and vertical dimensions of the material distribution in a sample surface.

## 1. Introduction

The effective path lengths and therefore attenuation of individual X-rays through a sample in an XRD (X-ray diffraction) or XRF (X-ray fluorescence) experiment depend on the local microstructure. This leads to absorption effects on the measured intensities. We summarize all sample characteristics which differ from an ideally packed, homogeneous and flat sample as microstructure. This includes surface roughness and porosity on different length scales as well as the spatial distribution of crystallites and the ratio of attenuation coefficients in multiphase samples. All these characteristics may occur in any combination. For precise data analysis it is important to know when corrections are needed, how severe the corrections will be and how the corrections depend on both the incident and scattering angle.

Harrison & Paskin (1964 ▸) showed how the absorption effect depends on the correlations between the paths of the X-rays in and out of the sample. They considered each individual path a diffracted X-ray takes through the sample explicitly, making their approach theoretically valid for any material distribution and measurement geometry. If there are no correlations between the beam paths, there is no angle-dependent absorption effect. Due to the complexity of mathematically describing realistic samples within their theoretical framework, Harrison & Paskin applied their theory only to a homogeneous solid with small and well separated pores without surface roughness.

To this day, the key problem is the incorporation of adequate mathematical models, representing real, non-ideal samples into the calculations of absorption effects. Real samples present a wealth of microstructural features on various length scales, both on the surface as well as in the bulk. A multitude of different simplifications and mathematical models has been used to describe real samples when calculating the effect of absorption on measured intensities.

Periodic, triangular (Borie, 1981 ▸) or rectangular (Masciocchi *et al.*, 1991 ▸) modulated 2D surface models allow the direct calculation of the absorption correction by explicitly considering each possible X-ray path through the sample analytically. For more complex models, explicit, analytical treatment of all X-ray paths is not feasible. Wilchinsky (1951 ▸) used a laminar model, separated into crystallite and grain layers, to describe a powder sample. Otto (1984 ▸) numerically analyzed the influence of inhomogeneity in a powder sample with a 2D model of equally sized circles representing individual particles. To describe the local 3D microstructure on the scale of a whole sample, complex statistical approaches are needed. Correlation functions can be used to describe the distributions of pores or grains within the bulk of a more realistic sample (Hermann & Ermrich, 1987 ▸; Hermann & Collazo, 1995 ▸) or to describe the height variations along its surface (Hwang & Houska, 1988 ▸).

The difficulty of detailed characterization of real, non-ideal samples aggravates the verification of any theoretical model. On the other hand, ray-tracing of individual X-ray paths through any specific sample model leads to an exact absorption correction. Simulation of models with variation of specifically chosen parameters provides insight into how certain microstructure features like roughness, waviness or porosity influence the angular dependence and magnitude of the absorption correction. Collazo *et al.* (1998 ▸) used a Monte Carlo ray-tracing simulation to study the influence of particle size on the absorption corrections for cylindrical samples measured in transmission geometry.

Here we present a practical algorithm for the simulation of absorption effects in XRD experiments in reflection geometry. The path of individual X-rays through the sample is traced and a transmission factor for every ray is calculated. The incident angle α and scattering angle 2θ can be chosen independently, allowing for simulations in symmetric as well as asymmetric scattering geometries. The material distribution within the sample is described by a 3D grid of voxels. Ray-tracing on voxel volumes has been discussed extensively in the context of X-ray tomography (Graetz, 2022 ▸), and we introduce here a modified shear-warp approach (Lacroute & Levoy, 1994 ▸). Arbitrary surface roughness, waviness, particle distribution and porosity can be modeled with this approach by adapting the voxel size to the geometrical complexity and fineness of the material distribution. Even samples consisting of multiple phases with different absorption contrasts can be simulated in order to gain insight into how microabsorption influences quantitative phase analysis experiments.

We confine the sample models simulated in this publication to single-phase models without porosity, focusing on the effects of surface modulations, namely roughness and waviness. As a proof of concept, we show that the simulation can exactly reproduce the analytically known corrections for ideal, flat samples and for the triangular modulated surface analytically analyzed by Borie (1981 ▸). We simulate absorption corrections for rectangular modulated surfaces as well, discussing predictions made by Borie in the light of our results. The numerical approach also allows us to study sample models which more closely represent the surface of typical powder samples, such as surfaces with pseudo-random height variations on different lateral scales created on the basis of Perlin noise. We demonstrate the importance of the lateral material distribution along the surface by showing its influence on the angular dependence of the absorption correction.

## 2. Fundamental principle

The fundamental principle of the simulation algorithm is the analytical calculation of the absorption of X-rays within a compact block of homogeneous material and then extension of the calculation to a sample made up of a large number of small blocks with possibly different materials. For each individual block the paths in and out of the sample are considered. The ray-tracing is realized by shifting the entire sample model with respect to the incident and diffracted angle before the calculation of the transmission factors. We assume a perfectly parallel incoming beam. Furthermore, we assume that each model is large enough to be a statistical representation of the microstructure of the sample. In this case, considering each individual beam path within the model gives an absorption correction that is statistically representative of the whole sample as well. We are working within the kinematic scattering theory, so multiple scattering is assumed to be negligible.

**Figure 1:**
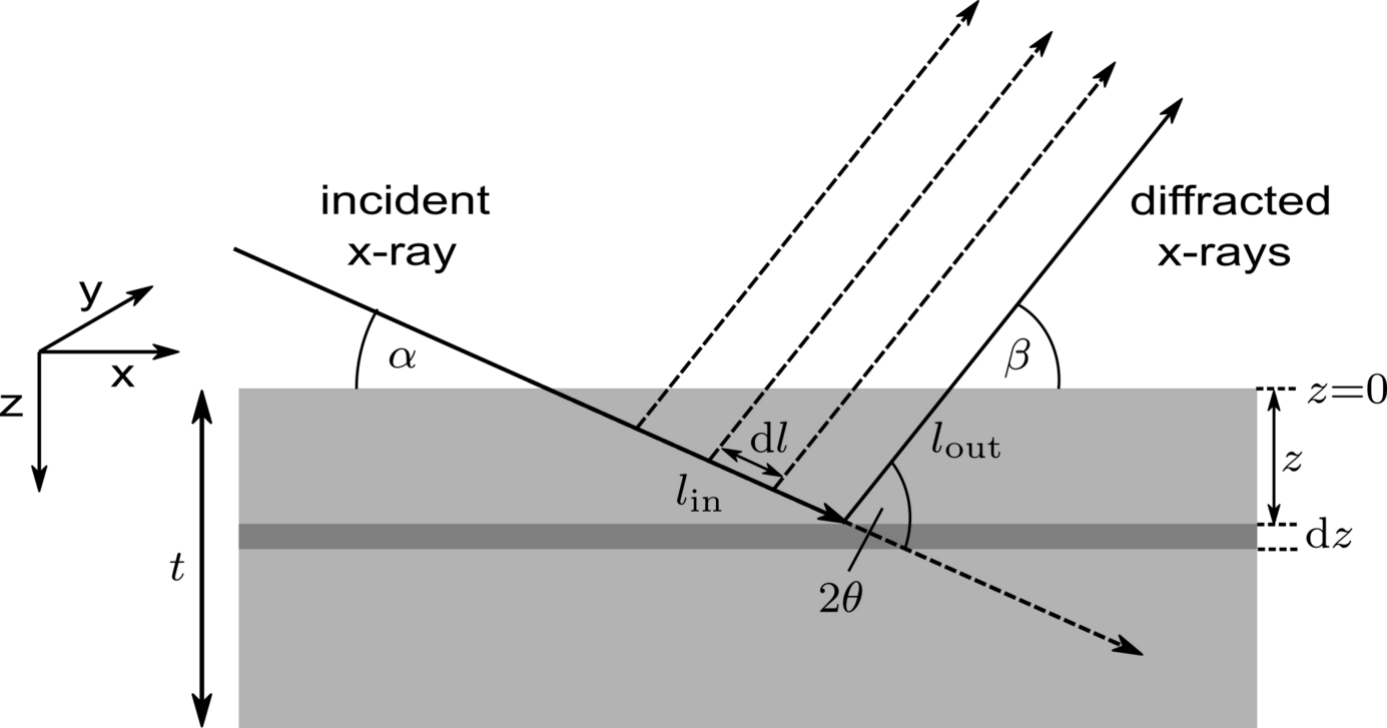
Beam path of an individual X-ray being scattered in depth *z* in a homogeneous, compact layer of thickness *t*.

Consider an individual X-ray hitting a homogeneous and flat sample at an incident angle α relative to the surface, as illustrated in Fig. 1 ▸. As the ray propagates along a path of length *l*_in_ through the sample, its intensity is attenuated according to Beer’s law by 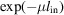, where μ is the linear attenuation coefficient of the material. Along the path of the X-ray a fraction of the intensity is diffracted with the diffraction angle 2θ. Each diffracted ray propagates out of the sample along the path *l*_out_ at an angle β = 2θ − α relative to the surface.

The linear attenuation coefficient summarizes absorption, incoherent and coherent scattering. For X-rays the absorption coefficient is much larger than the scattering coefficient. Because the difference between the attenuation coefficient and absorption coefficient is negligible, the linear attenuation coefficient is used before and after the scattering event.

The total path an individual ray has traveled through the sample before reaching the detector is *l*_in_ + *l*_out_. The fraction of intensity reaching the detector after the path *l*_in_ + *l*_out_ through the sample is given by the transmission factor 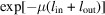. The total intensity observed at the detector is proportional to the sum of the transmission factors of all individual ray paths through the sample. In the limit of infinitesimal small path elements d*l* between scattering events this sum converges to the integral 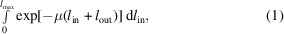
where *l*_max_ is the longest possible path into the sample.

Changing the integration variable to run over the depth *z* of the sample rather than along the incident ray d*l*_in_ leads to the expression of *l*_in_ and *l*_out_ as a function of the depth *z* in which the respective scattering event took place. The path out of the sample is always 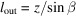. Substitution of 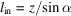 also changes the integration variable 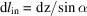. The integration then covers the whole sample thickness along the *z* direction, from 0 at the surface to the maximum thickness *t* of the layer,
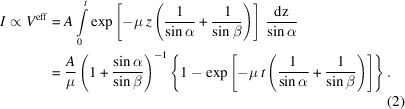
Here *A* is the cross section of the incident beam. The total intensity observed at the detector is proportional to the attenuation each ray of the incident beam experiences along its path through the sample. Therefore, the integration needs to cover the whole incident beam. Since we assume a perfectly parallel beam with homogeneous intensity distribution and the layer is homogeneous in both *x* and *y* directions, the total incident intensity is simply proportional to the cross section of the incident beam *A*.

**Figure 2:**
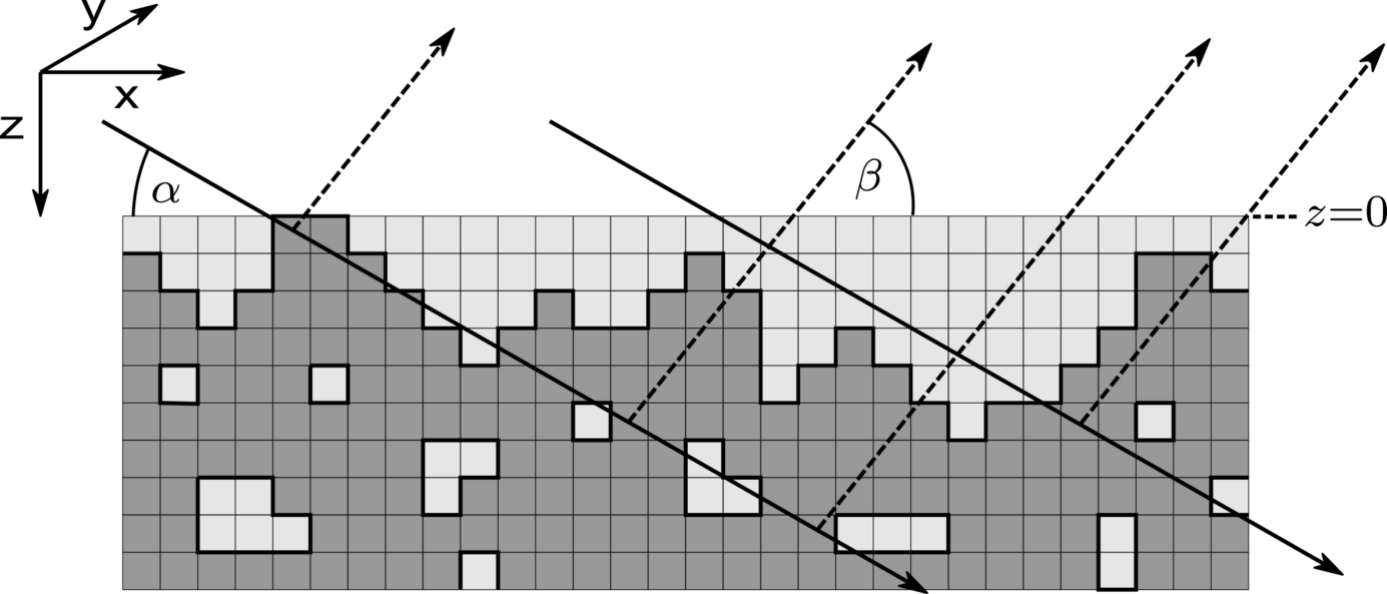
Model of a 2D slice of the material distribution in a single-phase sample exhibiting porosity and surface roughness. Incoming and diffracted X-rays experience significantly different attenuations depending on the location of the scattering event, which ultimately leads to absorption effects.

The proportionality factor *V*^eff^ is called the effective scattering volume. To obtain the absorption correction for any arbitrary measurement geometry, *V*^eff^ is calculated once for the measurement geometry of interest and once for the reference geometry with which the measurement should be compared, which is usually the symmetric geometry on an infinitely thick (*t* ≫ μ^−1^) and homogeneous sample. Substituting α = β = θ and taking the limit for *t* → ∞ of equation (2) results in the known, constant factor of 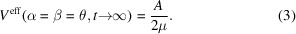
The absorption correction *C*_*a*_ is the ratio of both effective scattering volumes, describing how absorption changes the result of a measurement relative to the chosen reference (Rowles & Buckley, 2017 ▸),
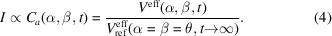


Equations (2) and (4) are well known results and describe infinitely thick samples and layer systems with compact, flat and homogeneous layers parallel to the surface. To extend the formalism to inhomogeneous samples, we need to consider that the attenuation along each individual ray path through the sample will be different depending on where the beam hits the surface and where the beam is diffracted. To model a sample with arbitrary surface roughness and porosity, the sample is divided into a 3D grid of equally sized, cubic voxels with edge length *d*, as illustrated in Fig. 2 ▸. Each voxel is either filled homogeneously with material or empty.

An X-ray will be attenuated by exp(−μ*l*) in each voxel the beamlet passes on the way in and out of the sample, where *l* is the geometric beam path within a voxel. Empty voxels do not contribute to the total attenuation and are assumed to be fully transparent to the radiation. Each diffracting voxel itself is compact and homogeneously filled with material, so its effective scattering volume 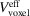 can be calculated with equation (2). The effective scattering volume of every individual voxel needs to be weighted with the attenuation of the X-ray beamlet along the total incident path to the corresponding voxel and the attenuation along the X-ray path after diffraction to the sample surface. The total effective scattering volume *V*^eff^ of the whole sample is then the sum over the weighted effective scattering volumes 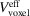 of all voxels. The absorption correction *C*_*a*_ can then be calculated with equation (4). This normalization will be discussed in detail in Section 3.1.

With this approach the absorption correction for any combination of incident and diffracted angle for any sample model can be calculated accurately, provided the microstructure of the sample is modeled with a sufficiently fine voxel grid. The limitations of the voxel grid resolution are discussed in detail in Section 4.3.

## 3. Technical implementation

The material distribution in a generally inhomogeneous sample is represented by a binary 3D array 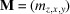 with *N*_*x*_, *N*_*y*_, *N*_*z*_ entries in the *x*, *y* and *z* directions. A filled voxel is represented by the numerical value 1, an empty voxel by 0. Each 2D cut along the *z* and *x* directions of the material distribution represents the sample in a single plane of incidence. Assuming a perfectly parallel beam in all dimensions, the incident rays propagate only in the *zx* plane. Therefore, the 3D problem can be treated as the average over multiple 2D slices through the material distributions. Consequently, any simulation of a 2D model will result in the same absorption correction as a 3D model which has a constant material distribution along the *y* direction. For illustration of the simulation algorithm, only 2D slices along the *z* and *x* directions are shown in the figures. The mathematical formalism is nevertheless described below for a 3D material distribution.

The incident, parallel X-ray beam is subdivided into *N*_*x*_*N*_*y*_ individual beamlets, one for each voxel at the surface of the material distribution. For each voxel *m*_*z*,*x*,*y*_ in the material distribution array **M** the corresponding transmission factor 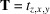 for an X-ray beamlet penetrating the voxel along path *l* is calculated as 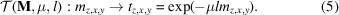
In the following arrays will always be denoted by a bold letter, while the element-wise defined operations resulting in the corresponding array are denoted with the respective calligraphic letter. In order to obtain the intensity reaching a voxel in depth *z*, the transmission coefficients of all penetrated voxels along the path towards that voxel are multiplied.

**Figure 3:**
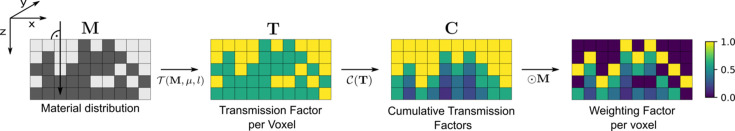
Illustration of the mathematical operations used to calculate the transmission factors for each voxel in the case of normal incident X-rays. In the first step an individual transmission factor for each voxel is calculated. Secondly, the transmission factors are cumulatively multiplied along the depth of the sample and shifted by one layer down to obtain the amount of intensity reaching a certain depth *z*. Finally, element-wise multiplication with the original material distribution **M** ensures empty voxels will have an effective scattering volume of 0.

First, we consider the special case of a beam with incidence and diffracted angles normal to the surface of the sample. The individual steps in the calculation are illustrated in Fig. 3 ▸. For normal incidence, the path *l* through each voxel in equation (5) is equal to the edge length *d* of a voxel. In this special case the cumulative product along the *z* direction of all the transmission factors results in another array **C**, where each entry *c*_*z*,*x*,*y*_ is the total transmission coefficient up to that voxel.

The beamlet reaching a voxel in depth *z* is only attenuated by the *z* − 1 voxels above. Since the incident beam hitting the first layer is not yet attenuated, the value in the first layer of **C** represents the incoming intensity 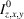 per individual X-ray beamlet. We assume all beamlets have the same, normalized intensity 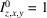, but generally any intensity distribution in the incident beam could be used. The transmission through the last layer is irrelevant, since the beamlet has transmitted the sample and no additional scattering event can occur. Formally, the array of cumulative transmission factors 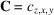 can be calculated element-wise from the array of transmission factors **T** as 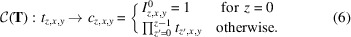


So far, we have only considered attenuation of the incident beamlets. The calculation of the attenuation from the voxel out of the sample follows the same principle, as the direction in which a voxel is penetrated is not of importance for the transmission factor. In the special case of normal incidence and backscattering α = β = 90°, the path of an X-ray beamlet to and from a diffracting voxel is identical, leading to identical arrays for the total transmission factors.

To get from the total contribution of a single voxel to the total intensity diffracted from the sample, the transmission factors for the incident and diffracted beamlet need to be multiplied together with the individual effective scattering volume per voxel 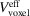. 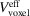 can be calculated via equation (2). As we are now considering a rasterized surface, the beam cross section *A* becomes the constant area of a voxel *A*_voxel_. Since the absorption correction will be calculated as a ratio of effective scattering volumes anyway [see equation (4)], the exact value of *A*_voxel_ is irrelevant. To take into account that empty voxels do not contribute to the scattered intensity, the transmission factors are multiplied with the original, binary material distribution. Finally, summation over all voxels results in the total effective scattering volume for α = β = 90°,
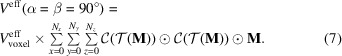
The symbol ⊙ describes element-wise multiplication of each individual element in the corresponding arrays.

**Figure 4:**
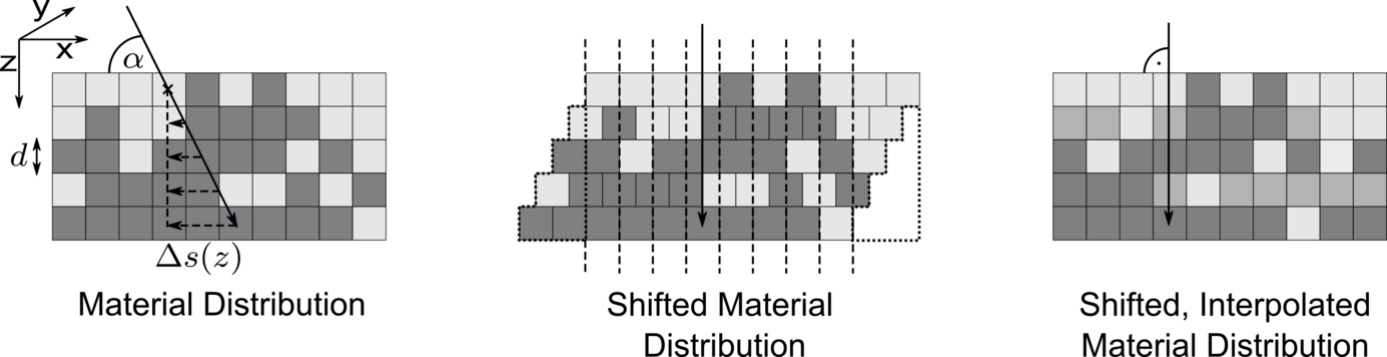
Illustration of the layer shift 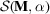 implemented to stack all voxels penetrated by the oblique incident beam in one column, in order to subsequently calculate the cumulative transmission coefficients along that path. We assume periodic boundary conditions.

To generalize the above concept to all scattering geometries, we consider a pair of incident and diffracted angles α, β ≠ 90°. The path of the beamlet through the sample will no longer be confined to only a single column. Instead of calculating the traversed amount of material along every oblique ray path through the sample, the whole material distribution can be shifted layer by layer in such a way that all voxels along the originally oblique path are stacked in a single column, as illustrated in Fig. 4 ▸. After the shift, the cumulative transmission factors **C** can be calculated with equation (6) analogous to the case of normal incidence.

For an arbitrary incident angle α the required shift Δ*s* in layer *z* is given by
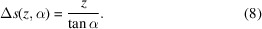
Generally, Δ*s* is not an integer value. Therefore, after the shift, two voxels may be partially located at the position of the original voxel, as depicted in Fig. 4 ▸. In order to account for these non-integer shifts, values in the new voxel are interpolated between both old voxels. Physically this corresponds to an averaging of the material content in the original voxels, which is consistent with the finite width of each beamlet. We assume periodic boundary conditions for the shift, meaning any voxels shifted out of the original size of the array are reinserted on the other side. With equation (8) the whole array shifting operation 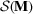 can be expressed element-wise as 
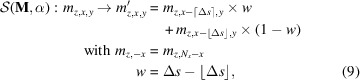
where 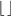 and ⌈ ⌉ denote the floor and ceiling function, respectively, and *w* is the linear interpolation weight.

**Figure 5:**
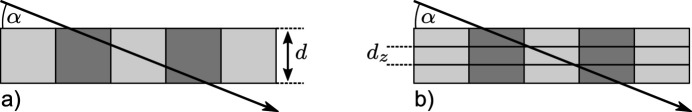
(*a*) X-ray beamlet passing through more than two voxels requiring (*b*) subdivisions to ensure the interpolation between two voxels is sufficient to describe the whole beam path within a single layer.

The procedure needs to be applied separately for the incident 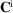 and diffracted 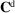 beam. Since α and β are defined with different rotation, the diffracted angle β needs to be adjusted to β′ = 180° − β in order to perform the shift obtained with equation (8) in the correct direction when calculating 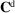. After the computation, the arrays need to be shifted back into the original coordinate system. Only then are the cumulative transmission factors correctly assigned to the voxel to which they correspond. With equations (5), (6) and (9)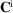 and 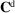 can be expressed as
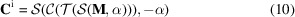

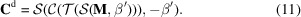
The arrays 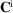 and 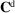 hold the total transmission factor an X-ray experiences along the path towards any voxel and from the voxel out of the sample, respectively. Consequently, the element-wise multiplication of both arrays results in the total transmission factor with which the effective scattering volume within the voxel needs to be weighted. However, not only does the material along the path of the X-rays change with the oblique angle, but also the path lengths through a given voxel. For any incident angle α or diffracted angle β, the path length *l* through each voxel changes according to 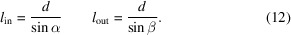
At angles smaller than 45°, the oblique path even traverses more than two voxels in a single layer, as illustrated in Fig. 5 ▸. The smaller the angle, the larger the amount of traversed voxels. Therefore, sublayers need to be introduced for angles <45°. Sublayers divide any given single layer of voxels into multiple layers with the same material content. The resulting voxels are no longer square, as their length along the *z* direction is reduced proportional to the number of new sublayers.

The amount of sublayers required is angle dependent as the sublayers need to be small enough to ensure that the oblique path through a given layer passes through at most two voxels. Thus, the voxel thickness *d*_*z*_ changes to 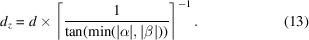
Since the calculation of 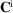 and 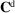 is defined element-wise, the calculation does not change for the subdivided arrays, only the new voxel thickness *d*_*z*_ needs to be taken into account. To guarantee that 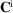 and 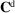 are of the same size for the subsequent element-wise multiplication, the subdivisions need to be equal and sufficient for both the incident and diffracted angle. Therefore, the smaller angle 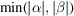 is used for the calculation of the number of required subdivisions.

Finally, the effective scattering volume of each voxel 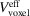 is calculated according to equations (12) and (13). 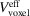 is then multiplied with the transmission factors in and out of the sample and summed up over the whole sample. Multiplication with the original material distribution **M** again ensures that empty voxels do not contribute to the sum. The result is the effective scattering volume of the whole sample, 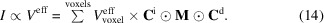


The above algorithm can be straightforwardly extended to models consisting of multiple phases by taking into account a binary material distribution for each phase. To calculate the transmission factors for multiphase samples, all phases need to be taken into account, since every voxel filled with material will attenuate a passing X-ray according to its phase-specific linear attenuation coefficient. The final summation of effective scattering volumes is done in a phase-specific way, since the diffraction is phase specific as well.

The ray-tracing of individual X-rays is handled with basic array manipulations only and is therefore easily accessible. It is implemented in Python/*numpy*. On conventional hardware [a desktop PC with an Intel Core i5-6500 processor (4 × 3.20 GHz) and 16GB RAM] the simulation runs on reasonable timescales. For example, a simulated measurement with 36 individual angle pairs for a model consisting of roughly 2 × 10^8^ voxels (see Fig. 14) takes just under 1 h. Since the attenuation of X-rays within each voxel is explicitly calculated, the limiting factor for the resolution of the voxel grid is the accuracy with which the microstructure of the sample can be modeled. Therefore, larger samples with larger microstructure features can be modeled with lower resolution without sacrificing the accuracy and precision of the resulting absorption correction, while simultaneously keeping the computational effort the same. The management of input and output data is handled via files in the .hdf5 format.

### 3.1. Calculating the absorption correction *C*_*a*_

The absorption correction *C*_*a*_ is the scaling factor which scales the measured intensity for a sample with arbitrary microstructure to the intensity expected in a symmetric measurement of the same material without microstructure. The measured intensity is proportional to the effective scattering volume calculated by the algorithm. As shown in equation (4), to obtain the absorption correction *C*_*a*_, the effective scattering volume *V*^eff^ for a measurement of a sample with microstructure needs to be normalized with the effective scattering volume 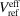 for a symmetric measurement of the flat and compact reference sample. Doing so isolates the absorption effects originating from the microstructure of the non-ideal sample. A complete measurement can be simulated by systematically varying the incident and/or diffracted angles and calculating the effective scattering volume for each pair of angles. Since *C*_*a*_ is a scaling factor, experimentally measured intensities need to be divided by *C*_*a*_ in order to obtain the corrected intensities without the influence of absorption.


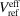
 can be obtained without having to explicitly model an ideal sample. For normal incidence and backscattering, the X-ray paths in and out of the sample are identical. Assuming an infinitely thick sample and no absorption in empty voxels, the effective scattering volume at α = β = 90° is independent of the microstructure of the sample and is always identical to that of a flat and compact sample. Since the effective scattering volume for flat and compact samples is also angle independent [see equation (3)], the absorption correction *C*_*a*_ can be calculated by normalizing the effective scattering volume at every angle of the measurement with the effective scattering volume at α = β = 90°.

Other normalizations are possible, since the effective scattering volume can be calculated for any measurement geometry and for any sample model. For example, asymmetric measurements can be scaled to symmetric ones on the same sample. Experimentally, measuring a sample in both symmetric and asymmetric conditions is typically easier than obtaining a perfectly flat reference sample, if a perfect reference sample can be obtained at all.

Our algorithm works for sample models with any thickness. The total effective scattering volume is naturally limited by the amount of material in the sample. Scaling the total effective scattering volume of a thin sample to the effective scattering volume of an infinitely thick (μ*t* ≫ 1) sample yields the thin film correction. Working with thin sample models which exhibit microstructure will of course lead to a correction *C*_*a*_ that respects both the influences of sample thickness and microstructure. In this case an additional simulation of an infinitely thick sample is required to obtain 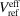.

## 4. Verification of the algorithm

To validate the programmed ray-tracing algorithm and its implementation, we simulate absorption corrections on simple sample models for which the exact analytical solution is known. Firstly, we show that the ray-tracing algorithm reproduces the absorption corrections calculated for a flat sample without any microstructure. Secondly, simulated absorption corrections for a periodically modulated, triangular sample model are compared with their exact analytical solution calculated by Borie (1981 ▸).

### 4.1. Simulations for samples without microstructure

The absorption corrections for flat, homogeneous and compact samples can be calculated analytically with equations (2) and (4). For comparison, the absorption correction for an equally flat, homogeneous and compact sample model can be calculated numerically with the ray-tracing simulation. The individual voxels in the sample model have an edge length of μ*d* = 5 × 10^−3^. An X-ray traveling perpendicular through a voxel of this length would lose about 0.5% of its intensity. Generally, the sample models used in this publication are ‘infinitely thick’ (μ*t* ≫ 1), making transmission effects through the whole sample negligible. Unless stated otherwise, we used in all simulations an intensity cut-off criterion of 10^−5^. If all individual beamlets reaching the detector have been attenuated at least to a factor of 10^−5^, the calculations are stopped. Only two measurements on the flat surfaces are simulated with thinner sample models to compare the results with the analytical thin film correction. Since we assume no divergence of the incident beam, it is sufficient to simulate the absorption correction for a 2D cross section of the sample model along the direction of the incident beam.

The absorption corrections are simulated for three different measurement geometries: the symmetric θ–θ geometry and two asymmetric measurement geometries where either α or 2θ are kept constant during the measurement and the respective other angle is varied. In practice, measurements of entire powder diffraction patterns with constant incident angle α are typically used for the analysis of thin film samples. Measuring a single Bragg reflection at variable incidence angles provides information about possible compositional depth profiles, texture or strain within the sample.

**Figure 6:**
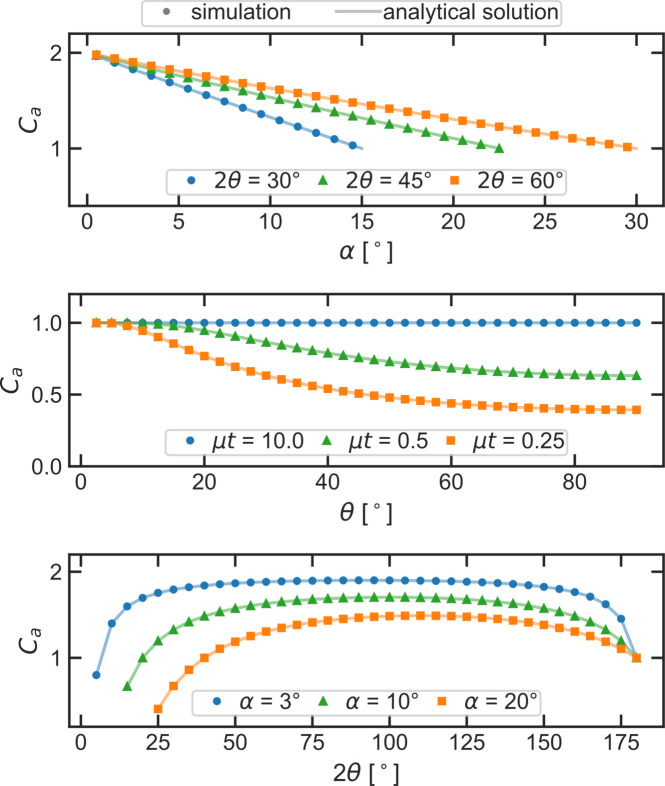
Comparison between analytical (solid line) and simulated (dots) absorption corrections *C*_*a*_ for three different measurement geometries for a flat, homogeneous and compact sample: (*a*) α scans at constant 2θ, (*b*) symmetric measurement with α = β = θ on samples with variable thickness μ*t* and (*c*) 2θ scans at constant α. If not specified otherwise, the simulated models were infinitely thick (μ*t* ≫ 1). The simulations reproduce the analytical results for all three geometries.

Fig. 6 ▸ shows the simulated and analytical absorption corrections for the three measurement geometries. For every geometry the simulation reproduces the analytical result exactly. The simulation of symmetric measurements on the thin sample models also reproduces the analytically calculated thin film correction.

### 4.2. Triangular modulated surfaces

**Figure 7:**
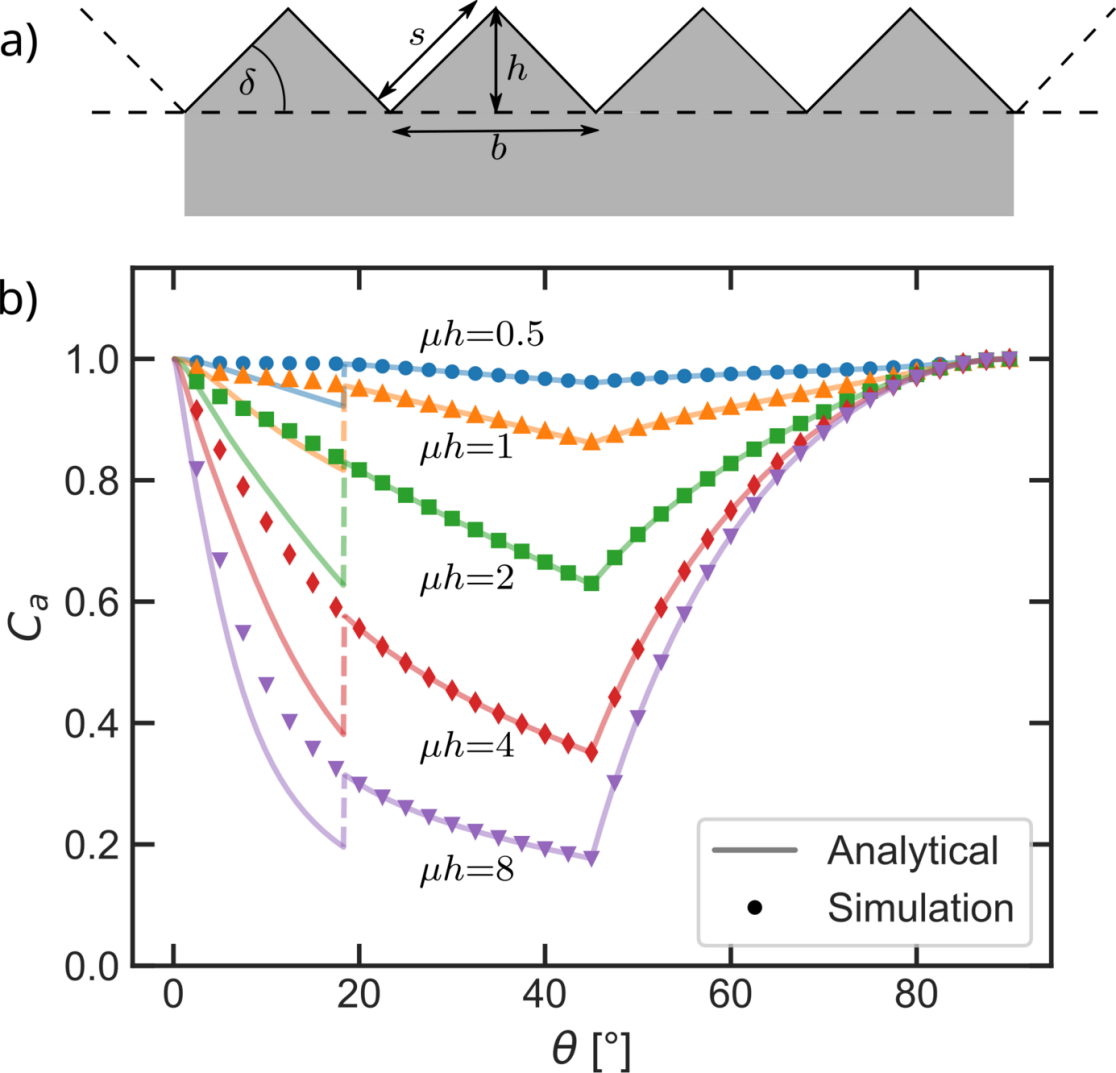
(*a*) Sketch of a surface modulated with isosceles triangles. It is sufficient to simulate one repeating unit due to the periodic boundary conditions. (*b*) Comparison between the analytical absorption corrections calculated by Borie (1981 ▸) (solid line) and the simulated absorption corrections (points) on a triangular modulated surface with a slope angle of 45° for variable heights μ*h*. The results are in good agreement apart from the discontinuity at θ = 18.4° which is discussed in the supporting information.

To show the validity of the ray-tracing we compare the simulated absorption correction with the exact analytical solution for a sample with microstructure. Borie (1981 ▸) calculated the exact absorption paths through a sample whose surface is periodically modulated by isosceles triangles with a slope angle δ = 45°. The slope angle is the angle between the flat surface normal and the slope of each triangle. The model is illustrated in Fig. 7 ▸(*a*). In his calculations Borie assumed symmetric measurements on an infinitely thick sample. The only variable parameter was the absorption-weighted height of the triangles μ*h*. In the context of absorption, length scales are only meaningful relative to the linear attenuation coefficient μ of the material, which is why the length scales of all models are given relative to μ.

In order to compare the simulation algorithm with Borie’s analytical solution, we modeled the triangular surface by a grid of voxels with edge lengths μ*d* = 5 × 10^−3^. The model is restricted to one repeating unit in 2D which is sufficient due to the periodic boundary conditions.

The simulated absorption corrections are in good agreement with the absorption corrections Borie presents in his publication, as illustrated in Fig. 7 ▸(*b*). The deviations between the calculated corrections and the simulated corrections for angles smaller than 18.4° result from the equations given in his paper, but are not present in the original plot of the corrections by Borie. The equations given by Borie (1981 ▸) are discussed in the supporting information.

### 4.3. Influence of model resolution

To assess the voxel size required to obtain accurate corrections, we simulated Borie’s triangular models with varying voxel lengths and compared how closely the simulated absorption corrections agree with the analytically calculated solution.

**Figure 8:**
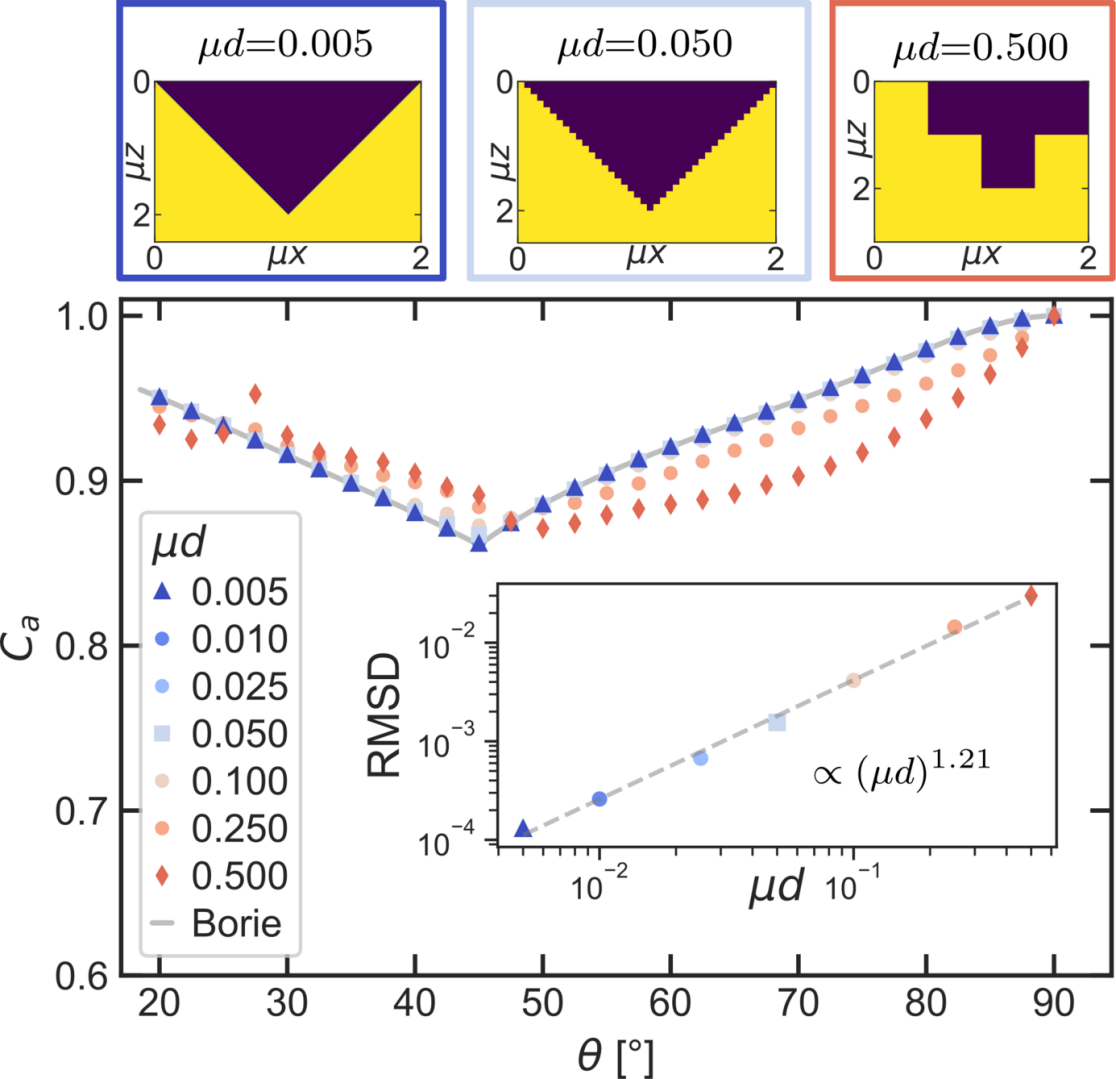
Simulations of a triangular sample model with μ*h* = 1 with varying voxel edge lengths μ*d*. The repeating unit of three of the models with significantly different voxel sizes is illustrated above the plot. In the inset the root-mean-square deviation (RMSD) between the simulations and Borie’s analytical solution is plotted over the voxel size.

The used voxel sizes range from the fine resolution used in the previous simulations of μ*d* = 5 × 10^−3^ to μ*d* = 0.5, a very coarse resolution which is technically no longer suitable to model a triangle. The absorption corrections for all resolutions are plotted in Fig. 8 ▸. We calculated the root-mean-square deviation (RMSD) for each voxel size, defined as 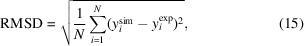
with the number of data points *N*, the value of the simulated data points 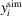 and the analytically expected values 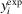. The comparison is limited to the angular range above 18.4° (see Fig. 7 ▸). The RMSD is fitted with a linear curve on a double logarithmic scale:
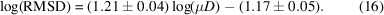
Comparing analytical and numerical solutions for each resolution shows that even moderate sampling produces results with less than 1% deviation from the actual analytical solution. Generally, the resolution of the sample models needs to be chosen relative to the size of the microstructure features of the sample. The relationship between voxel size and RMSD is nearly linear, as it can be described with a power law with exponent 1.21 ± 0.04.

The simulated absorption corrections and their analytical counterparts are in agreement with each other for both flat, compact samples and for Borie’s triangular surfaces. We conclude that the summation of the individual, weighted scattering volumes in each voxel and the shifting of the material distribution to calculate oblique-angled paths through the sample are valid.

## 5. Periodically modulated rectangular surfaces

**Figure 9:**
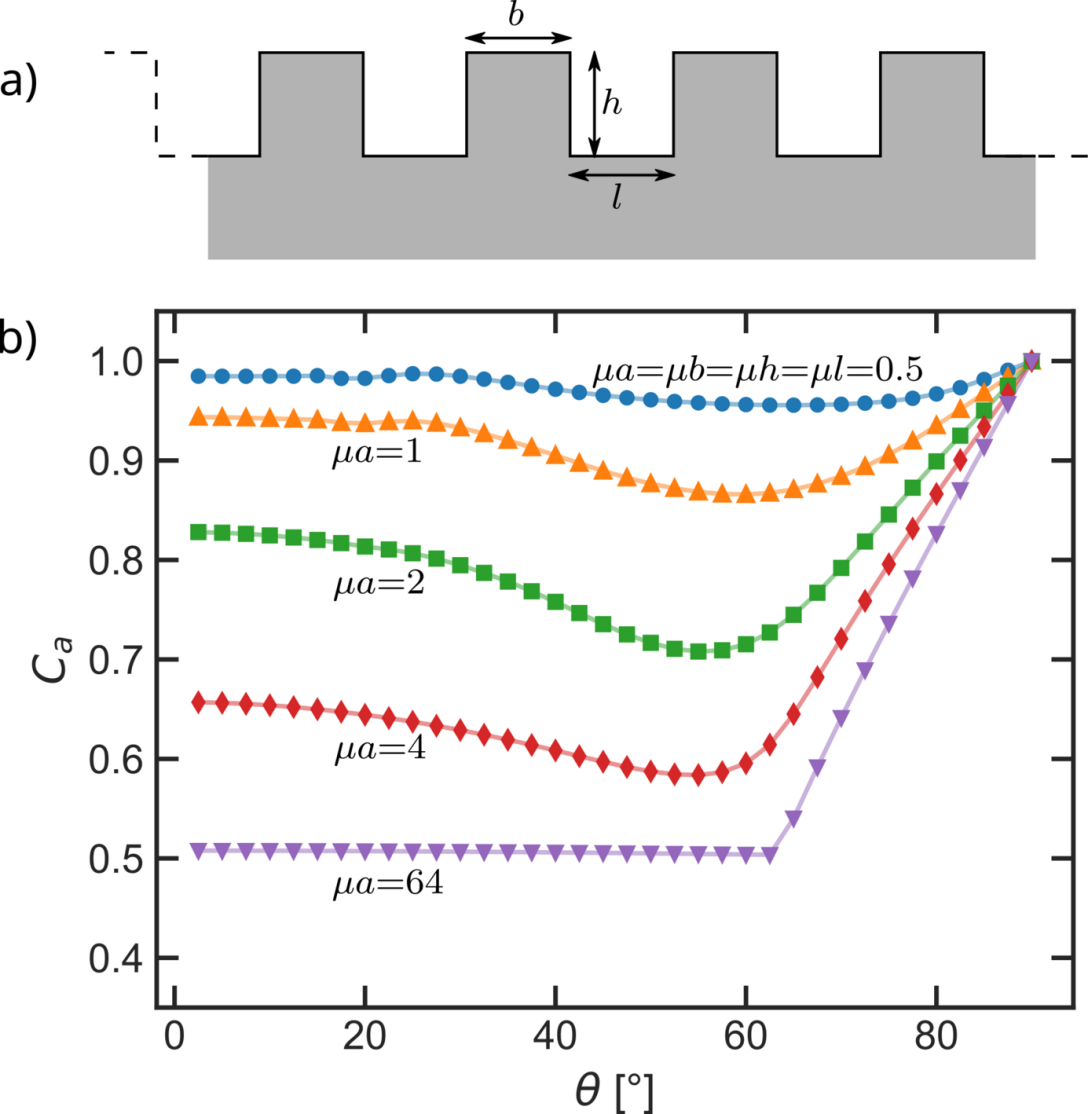
(*a*) Sketch of the rectangular surface model. (*b*) Absorption corrections *C*_*a*_ simulated for square-wave modulated surfaces with different edge length 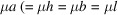). The lines through the simulated data points serve as a guide to the eye.

Borie predicts in his paper that the low-angle limit of the absorption correction would approach 0.5 for a square-wave modulated surface when the edge of the squares becomes long enough. Rectangular and therefore also square-wave surfaces in 2D are straightforward to model. The respective absorption corrections can be simulated and compared with the results for the triangular surfaces. Fig. 9 ▸ shows a sketch of a rectangular surface and its absorption corrections for the symmetric measurement geometry and a square-wave model. Here, only the edge length 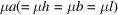 of the individual squares has been varied.

**Figure 10:**
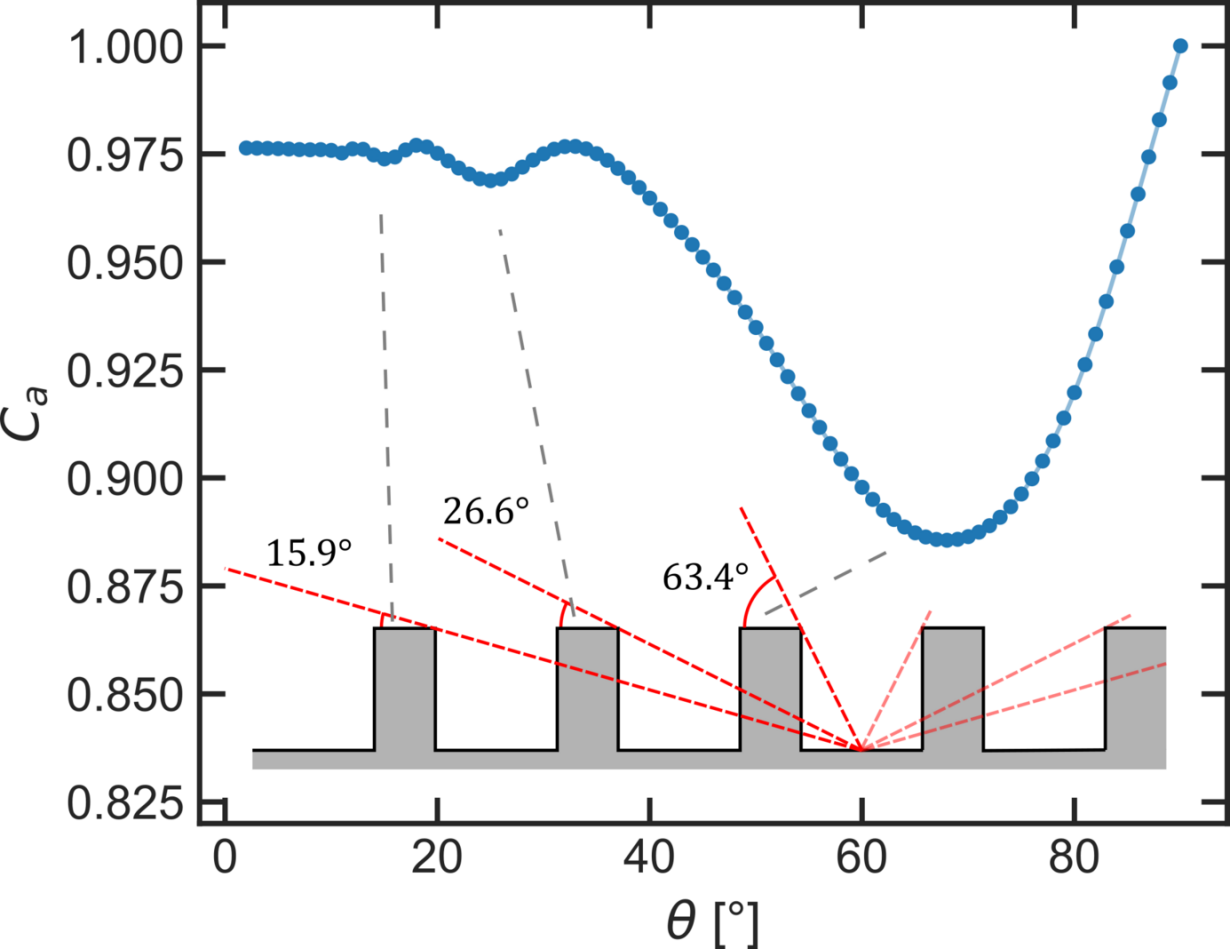
Illustration of the origin of higher-order minima in the absorption correction for a rectangular model with μ*b* = 0.5 and μ*h* = μ*l* = 1. If μ*b* is small enough, incident and diffracted X-rays may penetrate multiple rectangles before hitting the bulk, leading to the observed minima. The geometrically expected angles and the actual angle of the minima do not match exactly due to partial transmission of X-rays through the rectangles.

Similar to the triangular surface modulations, the minimum in the absorption corrections results from X-rays hitting the bulk of the sample model directly without being absorbed in rectangles beforehand. For a non-transparent material (μ → ∞) the theoretical minimum is expected at 63.4°. However, due to the transparency of the material the minimum is slightly shifted from the geometrically expected value, as X-rays with very short paths through the corner of a rectangle are hitting the bulk of the model already at slightly smaller angles. When μ*b* is small enough the X-rays may even penetrate one or more rectangles fully before hitting the bulk of the model, leading to ‘higher-order minima’ in the absorption correction at accordingly smaller angles, as illustrated in Fig. 10 ▸. This is of course an effect originating from the strict periodicity of these surface modulations. However, the higher-order minima demonstrate that the lateral material distribution on a scale larger than μ^−1^ is of importance for the absorption correction.

In contrast to the triangular surfaces the absorption corrections for square-wave surface modulations do not approach unity for small angles. Borie (1981 ▸) predicted that the absorption correction should approach 0.5 for large squares. For a square-wave surface model, 0.5 is the fraction of material in the topmost layer at *z* = 0. X-rays not hitting the top of a square are completely absorbed before they can exit the sample. The actual limit determined with our simulation for the square-wave surfaces is slightly higher than one half as there are always at least some X-rays which are diffracted at the side of the square and travel out of the sample through the corner of the rectangle.

**Figure 11:**
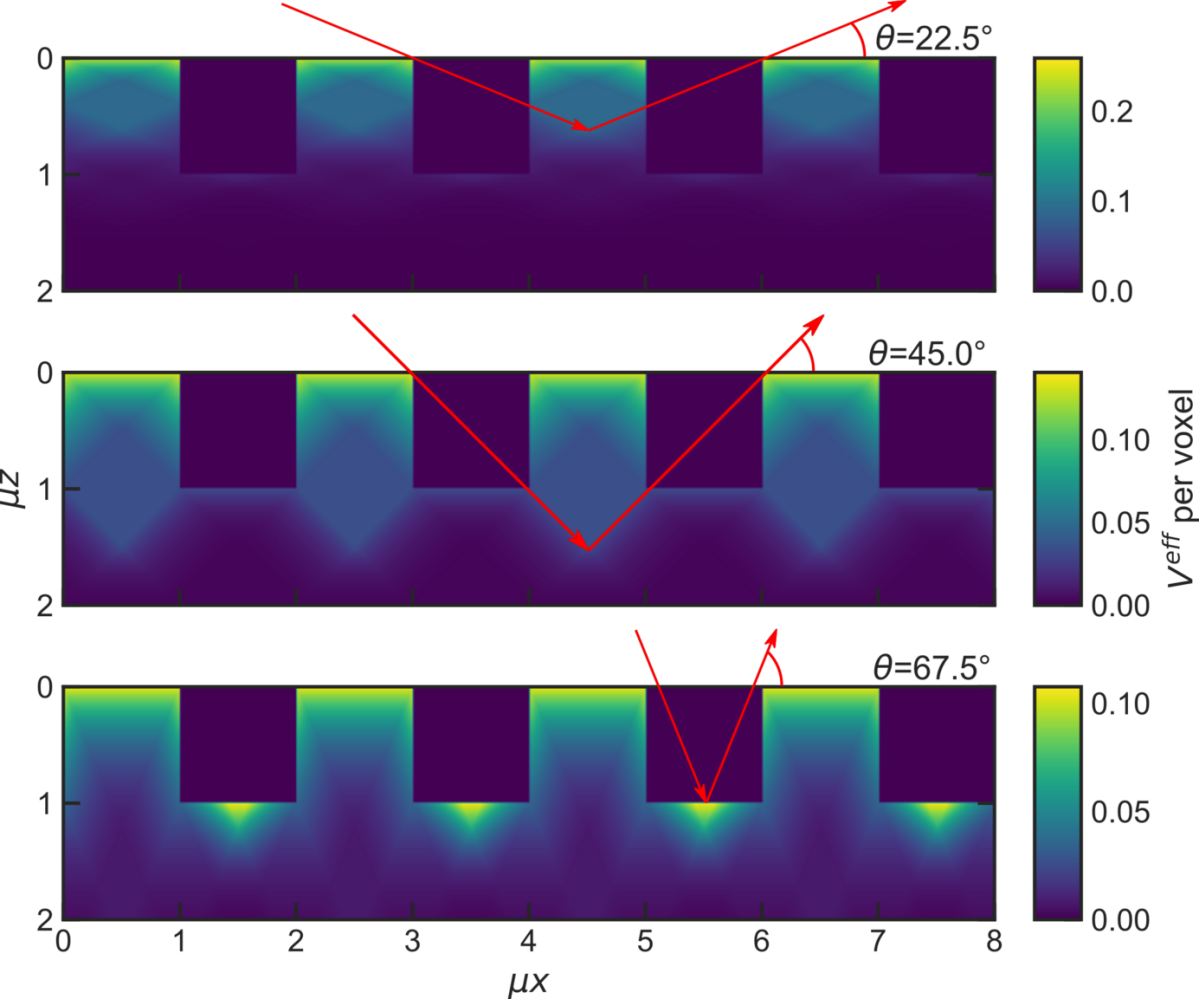
Distribution of the effective scattering volume for a square-wave modulated surface. The visualization shows how the parts of the sample contributing to the total effective scattering volume change with the angle and how the bulk of the sample only significantly contributes at angles high enough for the X-rays to leave the sample without having to go through a square on their way out.

These effects can be understood by visualizing where in the sample the scattered intensity comes from. Since the simulation algorithm calculates the effective scattering volume for every voxel of the sample model explicitly, it can also be used to visualize the contribution of each individual voxel to the total scattering volume. Such a visualization is generally helpful to understand the angular dependence of the absorption correction and is depicted for a square-wave surface in Fig. 11 ▸. Animations of how these contributions change with each simulated angle for both a rectangular and triangular surface can be found in the supporting information.

## 6. Influence of the lateral material distribution on the absorption correction

With the numerical approach of the simulation, the complexity of the analyzable models is not limited by the complexity of the exact analytical calculations. It is possible to calculate a precise absorption correction for any material distribution. The defining geometric parameters of a surface model can be systematically varied and their influence on the absorption correction can be analyzed. We analyze the importance of the lateral material distribution along the surface by simulating both simple periodic and pseudo-random surfaces with varying short-range roughness and long-range waviness.

### 6.1. Periodically modulated triangular surfaces

Due to the complexity of the calculations, Borie only calculated the exact absorption paths for isosceles triangles with 45° slope. In the spirit of Borie, we consider periodically modulated surfaces as a simplified type of roughness. The ray-tracing simulation can calculate the absorption correction for triangular models [Fig. 7 ▸(*a*)] with variable triangle shape and size. The influence of the geometric parameters defining the triangular surfaces on the absorption correction can be analyzed and allows conclusions to be drawn on the importance of the lateral material distribution along a surface.

**Figure 12:**
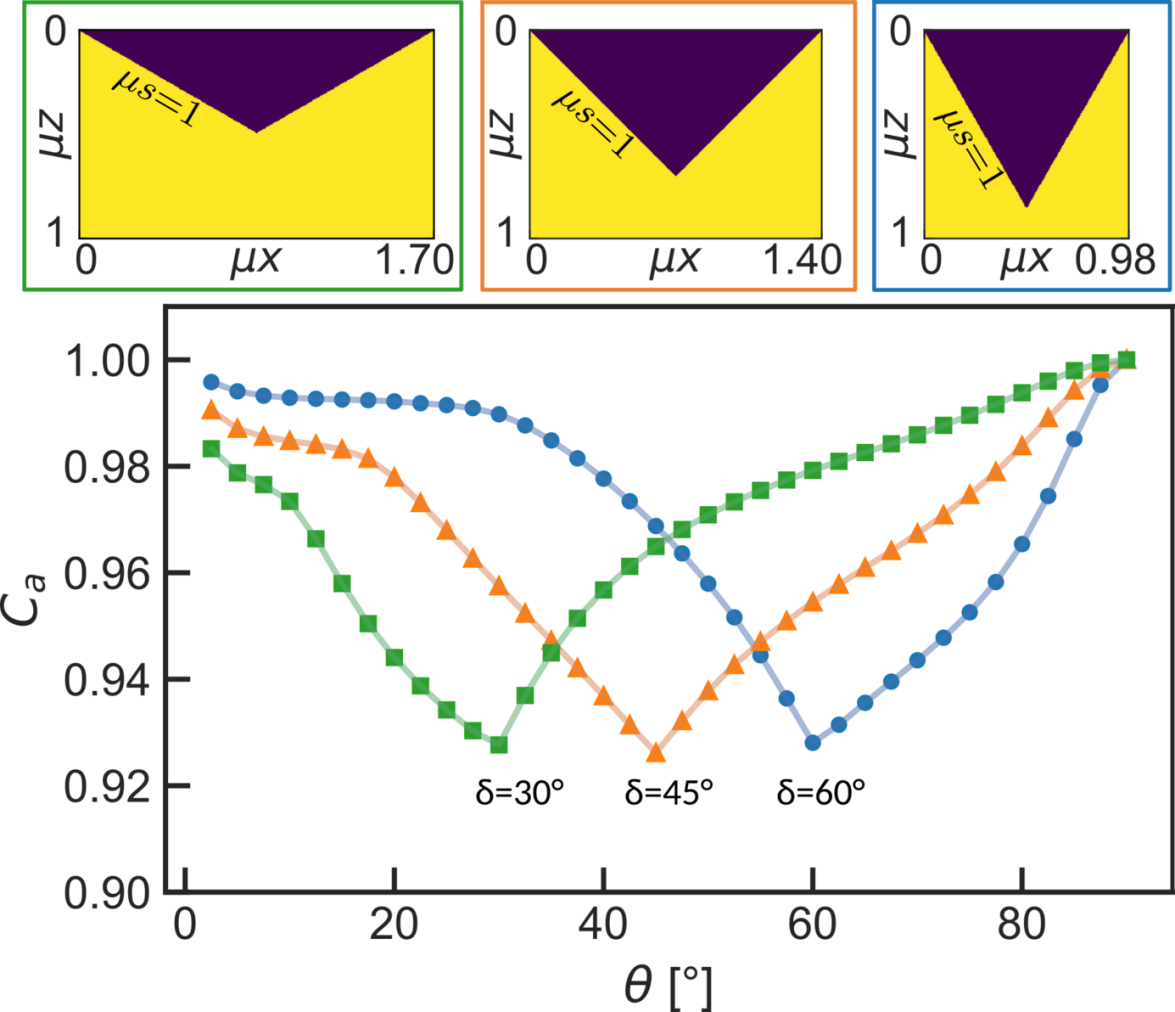
Simulated absorption corrections for triangular modulated surfaces with variable slope angle δ, but constant slope length μ*s* = 1. The lines through the simulated data points serve as a guide to the eye. The position of the minimum changes, while the value at the minimum stays constant within the accuracy of the simulation.

In Fig. 12 ▸ the absorption corrections for three models with constant slope length μ*s* = 1 and different slope angles δ are plotted. This translates into different *R*_pv_ values. The peak-to-valley distance *R*_pv_ is defined as the vertical distance between the highest and lowest point in the surface, regardless of lateral distance. The position of the minimum in *C*_*a*_ shifts to the respective slope angle δ. With constant slope length the value at the minimum is constant as well. The two defining properties for a periodic surface bound by isosceles triangles are the slope angle δ and slope length μ*s*, with δ defining the position and μ*s* defining the value of the absorption correction *C*_*a*_ at the minimum.

**Figure 13:**
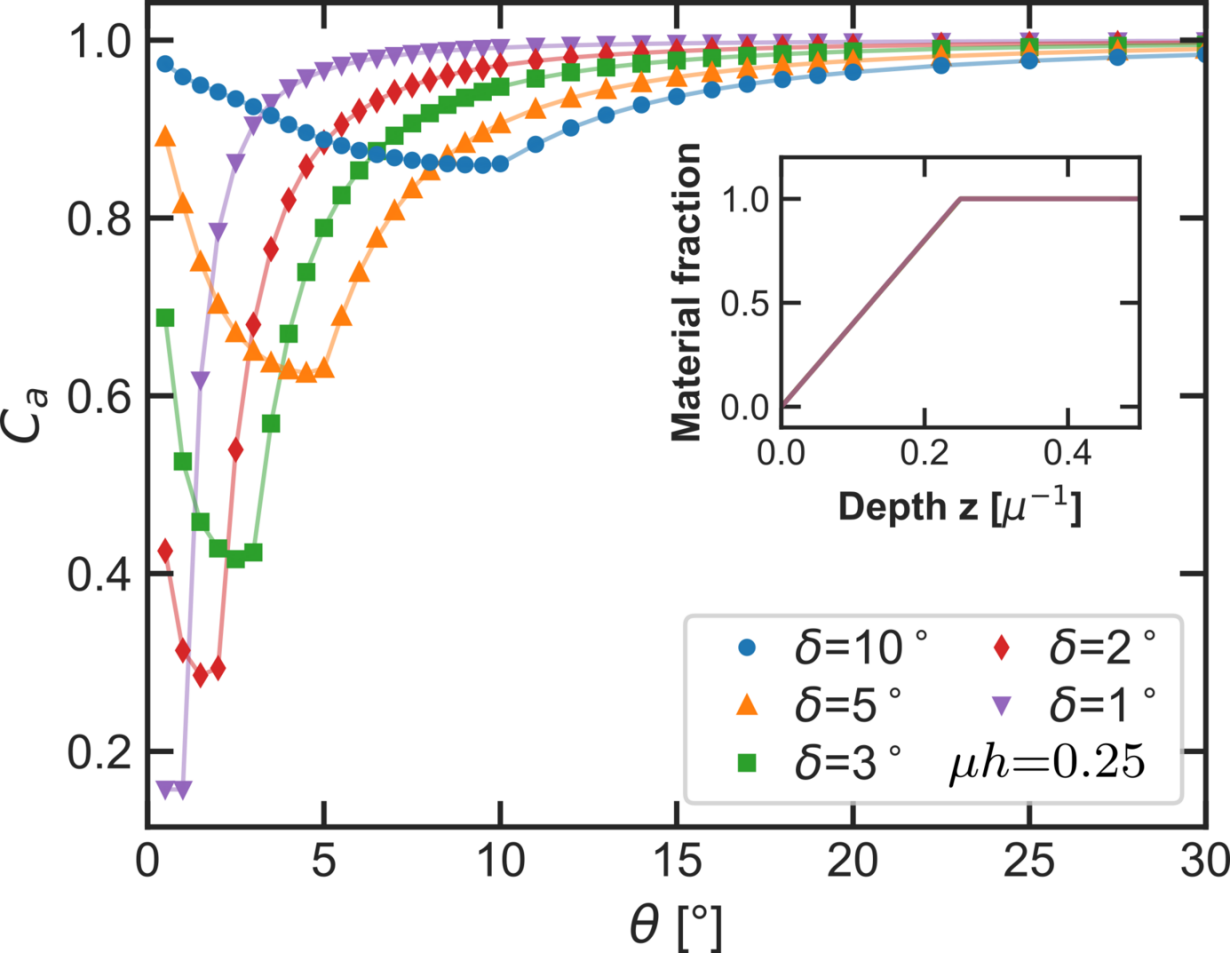
Simulated absorption corrections with constant triangle height μ*h* = 0.25 and varying slope angle δ. The inset shows that the material fraction over the depth does not change for these five models. Nevertheless, due to the change in slope length with the angle the minimum shifts to smaller values with decreasing slope angle. The lines through the simulated data points serve as a guide to the eye.

Triangular surfaces also allow us to model samples with identical height variations on different lateral scales in order to analyze the influence of the lateral material distribution on the absorption correction. Keeping the triangle height constant while changing the slope angle leads to a continuous transition from surface roughness to long-range surface waviness. Of course, the distinction between roughness and waviness in the context of absorption always needs to be made relative to the absorption length μ^−1^. The resulting absorption corrections in Fig. 13 ▸ show significant differences in their angular dependency. With decreasing slope angle, the minimum shifts to smaller angles as well. Due to the simultaneously increasing slope length, the minimum becomes deeper. The angular range in which the absorption corrections have a value significantly different from unity also becomes smaller with decreasing slope angles. In the limit of very small slope angles the absorption correction consequently approaches the constant correction expected for flat samples, as the sample model gets increasingly closer to a flat surface.

### 6.2. Pseudo-random 2D surfaces

The surfaces of samples typically used in powder diffraction experiments are not strictly periodic, but have randomly distributed height variations on varying lateral scales. Simple, periodic 2D sample models provide analytical access to the exact absorption correction. However, one may argue that the strict periodicity may also give rise to features in the corrections which are not to be expected for a surface with random material distribution. The simulation approach enables us to calculate a correction for arbitrary sample models, provided they are modeled with sufficient accuracy on a voxel grid. We created models with pseudo-random surfaces with Perlin noise (Perlin, 1985 ▸, 2002 ▸) as a basis for the material distribution. With this approach we can compare absorption corrections simulated on samples which exhibit non-trivial surface features on varying lateral and vertical scales.

**Figure 14:**
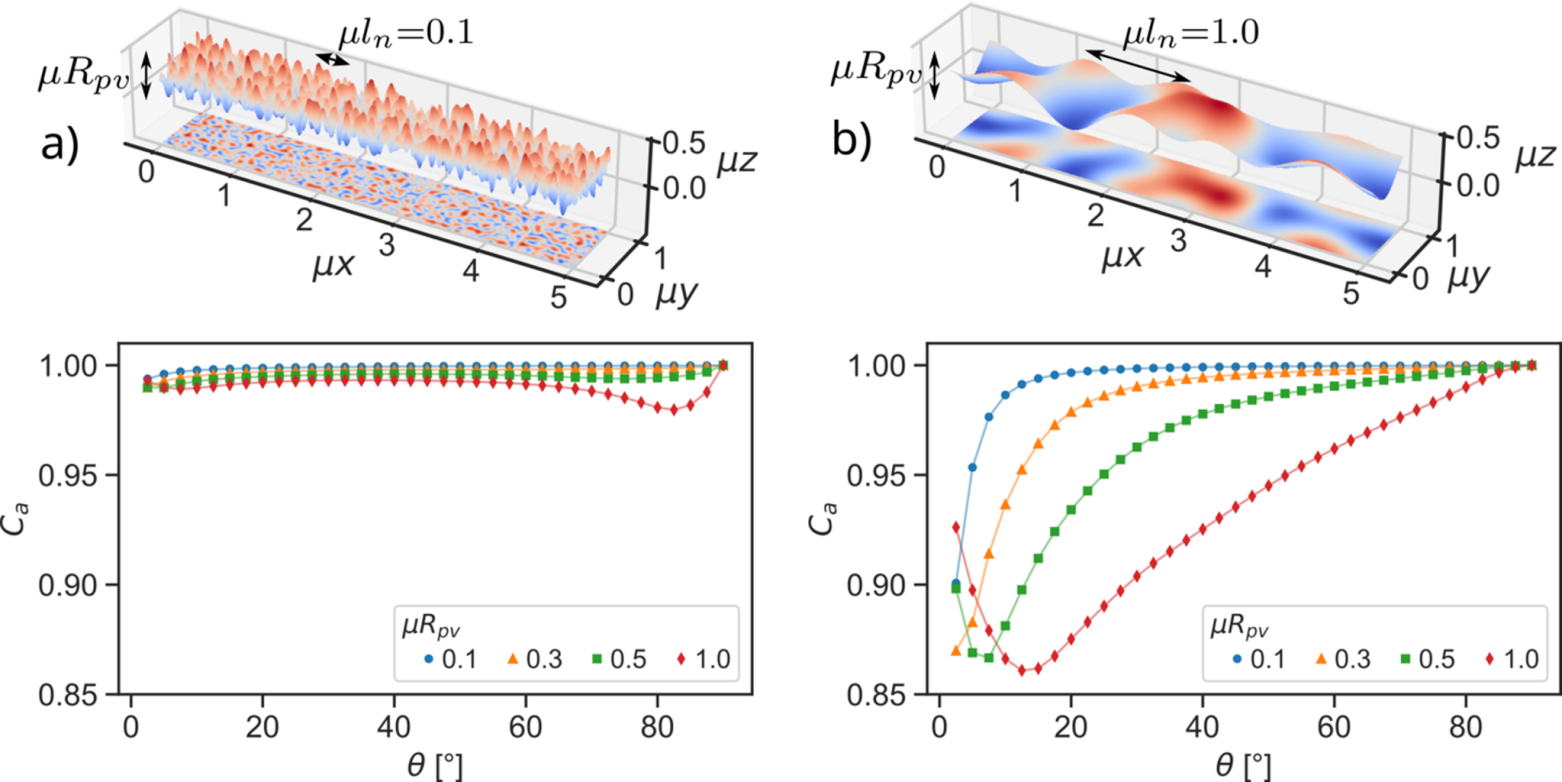
Comparison between the absorption corrections for (*a*) rough and (*b*) wavy sample surfaces created by mapping a 2D image of pseudo-random Perlin noise to different *R*_pv_ values. All modeled samples were infinitely thick (μ*t* ≫ 1) and had a voxel edge length of 5 × 10^−3^. The solid lines through the data points serve as a guide to the eye. The lateral distribution of material heavily influences the resulting absorption corrections. While the rough surfaces show minimal corrections, the wavy surfaces exhibit a strong angular dependency and a significantly larger deviation from unity.

To model samples with pseudo-random surfaces as seen in Fig. 14 ▸, first a 2D image of Perlin noise is generated [implementation in Python from Vigier (2019 ▸)]. The noise is used as height data for the final 3D model. Multiplication of the normalized noise with a scaling factor changes the range of the height variations of the resulting surface. The used scaling factor simultaneously defines the peak-to-valley distance *R*_pv_ of the surface. The cubic gradient grid used to create the Perlin noise defines the lateral scale of the surface modulations and is described by the distance between its nodes μ*l*_n_. We differentiate between roughness and waviness by the lateral scale of height variations in the surface relative to μ^−1^. A fine gradient grid with lateral scales much smaller than the attenuation length 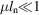 results in a rough surface. On the other hand, surfaces with lateral scales approaching or exceeding μ*l*_n_ ≥ 1 are referred to as wavy. As implied by our algorithm, all models were created with periodic boundary conditions in the *x* direction, explicitly avoiding inadvertent discontinuities.

We used normalized 2D Perlin noise with a fine gradient grid μ*l*_n_ = 0.1 to create a set of models which exhibit a different range of height variations *R*_pv_ on an identical lateral scale. This corresponds to a rough surface without waviness. The rough surfaces and the resulting absorption corrections are depicted in Fig. 14 ▸(*a*). Only the surfaces are shown, but all models are thick enough to neglect any transmission effects. The simulated absorption corrections show no strong angular dependence, especially in the range of a typical symmetric experiment (10°–140° 2θ). All absorption corrections are close to unity, even if the height variations *R*_pv_ are as large as the absorption length. The small offset from unity is proportional to the *R*_pv_ value of the surface.

Similarly to the rough surfaces, surfaces exhibiting waviness with variable *R*_pv_ can be modeled by using an image of Perlin noise with a larger gradient grid μ*l*_n_ = 1. The simulated absorption corrections have a strong angular dependency and show significant differences depending on *R*_pv_. Even at μ*R*_pv_ = 0.1, the lateral distribution of the material leads to a significant correction at small angles which quickly approaches unity at larger angles. The larger the ratio between lateral distances μ*l*_n_ and vertical distances μ*R*_pv_ gets, the smaller is the angular range in which the correction is significant. Surfaces with more pronounced height variations on the same lateral scale produce absorption corrections which differ from unity over the whole angular range from θ = 0° to θ = 90°.

### 6.3. Pseudo-random surfaces exhibiting roughness and waviness

**Figure 15:**
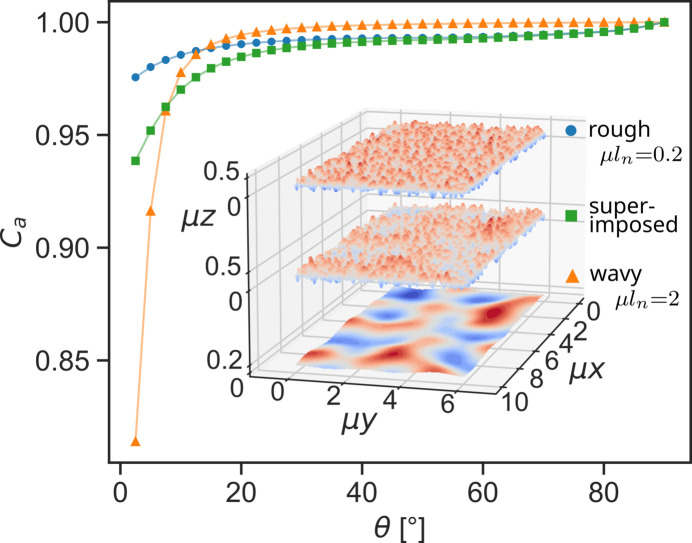
Model and resulting absorption correction created by the superposition of two images of Perlin noise with different gradient grids. The finer gradient grid has a node distance of μ*l*_*n*_ = 0.2 and μ*R*_pv_ = 0.5 while the long-range grid has a node distance of μ*l*_*n*_ = 2 and *R*_pv_ = 0.2. The voxel size of the model is μ*d* = 0.01. The absorption correction of the combined surface shows features observed for the purely rough and purely wavy surface.

Multiple instances of Perlin noise created on different gradient grids can be superimposed, to create a sample model with both short-range roughness as well as long-range waviness along its surface. Generally, it can be expected that real powder samples exhibit height variations on multiple lateral length scales. The degree of these variations depends of course on the particle size distribution and on the method of preparation. Such a surface with combined roughness and waviness is depicted in Fig. 15 ▸.

Over most of the angular range, specifically at higher angles, the absorption correction is nearly identical to that of the purely rough surface, which also shows the same offset from unity as the combined surface. As already indicated by the previous simulations, the waviness leads to an increase of the absorption correction at small angles. The same effect is observable for the combined surface, although the effect of the waviness is damped by the roughness. The surface exhibiting pure waviness without short-range roughness shows a more pronounced increase of the correction values in the low-angle regime. Nevertheless, the absorption correction of the combined surface clearly reveals influences from both its roughness and its waviness even though the waviness alone has smaller height variations than the roughness.

## 7. Discussion

With the simulation approach we are able to analyze the influence of microstructure features on the absorption corrections in symmetric diffraction geometry. Simulations for the triangular models in Fig. 12 ▸ show that the magnitude of height variations in the surface of a sample alone do not define the magnitude of the resulting absorption corrections. Instead, the combination of the vertical scale μ*h* and the lateral scale μ*b* of the triangles, namely their slope length μ*s*, defines the value of the minimum in the correction. A surface description based solely on the vertical scale of the surface along the *z* axis of the sample is insufficient to model absorption correctly. This is further highlighted by simulations for the triangular surfaces in Fig. 13 ▸, which all have the same peak-to-valley distance μ*R*_pv_ = μ*h* = 0.25, yet show significant differences in their simulated absorption corrections when the lateral extent of the triangle base changes. For these triangular models the average distance to the mean height *R*_*a*_ and the material fraction per depth is per definition the same as well (see inset in Fig. 13 ▸).

Harrison & Paskin (1964 ▸) describe the absorption effect with the correlation of the X-ray paths in and out of the sample. There is no absorption correction if the paths are entirely uncorrelated. On the compact samples analyzed here, the degree of correlation between the X-ray paths in and out of the sample is strongly influenced by the lateral material distribution. If the height varies on a lateral scale much smaller than the absorption length μ^−1^ the individual paths in and out of the sample have very little correlation. Consequently, the sample models with purely short-range roughness in Fig. 14 ▸(*a*) show small correction terms, which are almost constant over much of the angular range, at least relative to the precision of intensity measurements in powder diffraction. Any correction that shows no or a very weak angular dependence in the range of a measurement is indistinguishable from a scaling factor and does not change the relative intensity of measured Bragg reflections. On the other hand, on wavy surfaces the lateral scale of height variation approaches or exceeds μ^−1^ and thus the X-ray paths in and out are increasingly more correlated. As shown in Fig. 14 ▸(*b*), the surfaces exhibiting pure long-range waviness show significantly larger corrections and a stronger angular dependence than the surfaces with short-range roughness alone. This effect is also observable for the periodically modulated triangular surfaces (Fig. 13 ▸).

The correlations between the incoming and outgoing X-ray path also explain the angular dependency of the correction. For a sample without pores and a surface with pronounced waviness, the paths in and out of the sample are less correlated the higher the incident and diffracted angle θ becomes, leading to the asymptotic approximation of the correction to unity. The closer incident and diffracted angles are to θ = 90°, the smaller the distance between the entry and exit point of the X-ray on the surface becomes, meaning the X-ray experiences less of the lateral height variation. At θ = 90° the X-rays probe a locally ‘flat’ surface independent of surface modulation. Conversely, the smaller the incident and diffracted angle become, the larger the distance between the entry and exit point of the X-ray, meaning the height variation on a longer scale becomes relevant for the absorption effect. Approaching the limit of vanishingly small height variations μ*R*_pv_ → 0 over increasingly large lateral distances μ*l*_n_ → ∞, the angular range in which the correction differs from unity will become insignificantly small, as the sample surface approaches an ideal, flat surface [Fig. 14 ▸(*b*), μ*R*_pv_ = 0.1].

Theoretically, waviness on a lateral scale ≫μ^−1^ has no direct influence on the absorption correction. If the path through the sample becomes large enough, basically all intensity is fully absorbed before the X-rays leave the sample. In this case, the correlation between the path in and out is irrelevant for the absorption correction, since these X-rays do not contribute to the total measured intensity. However, it needs to be noted that large-scale waviness may very well also lead to correlations on the scale of the attenuation length, which will have an influence on the absorption correction.

In symmetric diffraction measurements, a pronounced minimum in the absorption is observed, when the surface exhibits holes with dimensions in the order of magnitude of the attenuation length. These holes provide paths for X-rays to reach points in the depth of the sample surface from where they are significantly absorbed on their way out. When incident and diffracted angles get large enough for X-rays to be scattered in these holes and leave the sample again, the scattered intensity increases. Before that point the observed intensity decreases, since with increasing incident angle more X-rays reach depths out of which they cannot leave again, effectively leading to the observed minimum. The visualization of the distribution of the effective scattering volume for the rectangular surface in Fig. 11 ▸ shows this effect (an animated version can be found in the supporting information). The corrections *C*_*a*_ for surfaces with μ*R*_pv_ ≥ 0.5 in Fig. 14 ▸(*b*) also show a minimum, which stems from both the lateral and vertical scale being in the order of magnitude of the attenuation length. It is reasonable to assume that such surfaces may result from powders which have particles with mean cord lengths >μ^−1^. On the rough surfaces the same effect can be observed to a smaller degree at high angles in Fig. 14 ▸(*a*) for the surface with μ*R*_pv_ = 1. Due to the large *R*_pv_ the holes become large enough as well, even for short-range roughness.

A surface with combined roughness and waviness shows a combination of features in its absorption correction as they are observed for purely rough and purely wavy surfaces. As seen in Fig. 15 ▸, the correction on the combined surface increases at low angles less than on the purely wavy surface. The local roughness damps the effect of the waviness.

We can qualitatively compare the absorption corrections obtained with the simulations with earlier published corrections determined experimentally with both XRF and XRD measurements. Because the energy of X-rays typically changes in XRF experiments, the attenuation of the X-rays on their way in and out of the sample changes as well. While the simulation in its current state is not designed to work with different attenuation coefficients for the path in and out of the sample, we believe qualitative comparison between corrections obtained from XRF measurements and the simulated corrections can still be made.

The simulated absorption corrections on the surface with superimposed roughness and waviness qualitatively agree well with the corrections observed in XRF measurements by Paakkari & Suortti (1968 ▸). Suortti (1972 ▸) further investigated the absorption effect in XRF measurements and separated his resulting corrections into an angle-dependent part originating from surface roughness and a constant part originating from porosity. Later, Hermann & Ermrich (1987 ▸) derived an analytical absorption correction based on a statistical model with the same separation of porosity and roughness effect and applied it successfully to Suortti’s data. We observe both the constant effect ascribed to porosity and angle-dependent effect attributed to the surface for sample models without porosity. In the simulated corrections, the features stem from roughness and waviness, respectively, revealing the lateral scale of height variations as the essential parameter to differentiate both effects.

The data measured by Trucano & Batterman (1970 ▸) in scattering experiments show a minimum in an angular range between 20° and 50°, similar to the corrections calculated by Borie (1981 ▸) for a triangular surface. The simulated absorption correction for the pseudo-random Perlin surface with both lateral and vertical scale in the order of magnitude of the attenuation length [Fig. 14 ▸(*b*)] most closely resembles those corrections, which is reasonable since Trucano & Batterman (1970 ▸) analyzed spherical glass particles with diameters μ*D* ≥ 1. A similar minimum is present in the analytical correction by Hwang & Houska (1988 ▸), who used a statistical model to describe the lateral distribution of material along the surface to derive their correction. With this approach they were able to describe their experimental XRD data. Pitschke *et al.* (1993 ▸) measured scattered intensities in XRD experiments on strongly absorbing tungsten carbide and YBa_2_Cu_3_O_7_ powders prepared by sedimentation in ethanol. The resulting corrections show a strong angular dependency over all of the angular range and reach a minimal value of 0.4 in the case of YBa_2_Cu_3_O_7_. Following the trend observable in the simulations of the pseudo-random Perlin surfaces in Fig. 14 ▸, these measured absorption effects point to a sample surface with significant waviness and roughness on a scale > μ^−1^. Both the reported mean cord lengths of the particles and the average surface roughnesses *R*_*a*_ are in the order of magnitude (tungsten carbide) or significantly larger (YBa_2_Cu_3_O_7_) than their respective attenuation lengths, leading to the observed magnitude and angular dependency of the absorption effect on these samples. On the other hand, the same powders prepared with a smooth surface by pressing them against a polished plate show no significant correction over most of the angular range of the measurements.

Despite their differences, the wealth of measured absorption corrections can be reproduced qualitatively with simulations for compact sample models with various surfaces. This highlights that absorption effects are highly dependent on the detailed microstructure of the sample.

## 8. Conclusion

We present a ray-tracing simulation to calculate the influence of microstructure on scattered intensities in XRD experiments in reflection geometry. We reduced the ray-tracing procedure to a sequence of elementary array resampling and multiplication steps, enabling the numerical computation of absorption corrections for arbitrarily complex structures. Considering the attenuation within individual voxels analytically makes the rasterization of the microstructure the limiting factor for the precision of the result. This allows the use of lower resolutions to model larger microstructure features on larger samples. The simulations can be carried out on conventional hardware in reasonable times. Furthermore, the calculation of the effective scattering volume per voxel enables the user to visualize the contribution of each voxel to the total scattering volume, giving insight into the origin of the scattered intensity. The simulation is applicable to non-symmetrical geometries as there are no restrictions with regard to incident and diffracted angles.

The simulations allowed us to numerically calculate absorption corrections not only for various simple periodic 2D surface modulations, but also for 3D models exhibiting pseudo-random surfaces with varying vertical and lateral extent of their material distribution. The simulations demonstrate that for compact samples the lateral scale of the material distribution in the surface relative to the attenuation length μ^−1^ is essential for the angular dependency and magnitude of the resulting absorption correction. Generally, absorption corrections become significant when the lateral scale of the microstructure approaches or exceeds the attenuation length. Surface descriptions based solely on height variations normal to the surface are insufficient to include all aspects of absorption effects.

The flexibility of simulating arbitrary sample models also allows the analysis of porous samples in the future. Furthermore, absorption corrections for real samples can be calculated after their microstructure has been analyzed experimentally with *e.g.* an atomic force microscope or an optical profilometer.

## Supplementary Material

Visualization of the distribution of effective scattering volume for a rectangular modulated surface. DOI: 10.1107/S2053273324003292/wo5048sup1.gif

Visualization of the distribution of effective scattering volume for a triangular modulated surface. DOI: 10.1107/S2053273324003292/wo5048sup2.gif

Full caption for rectangular gif. DOI: 10.1107/S2053273324003292/wo5048sup3.txt

Full caption for triangular gif. DOI: 10.1107/S2053273324003292/wo5048sup4.txt

Borie's analytical equations. DOI: 10.1107/S2053273324003292/wo5048sup5.pdf
