# Supplementary figures and images for: Universal simulation of absorption effects for X-ray diffraction in reflection geometry
Source: Acta Crystallogr A Found Adv. 2024 Jun 7;80(Pt 4):315–28. doi: 10.1107/S2053273324003292 (PMC11216610; doi:10.1107/S2053273324003292)

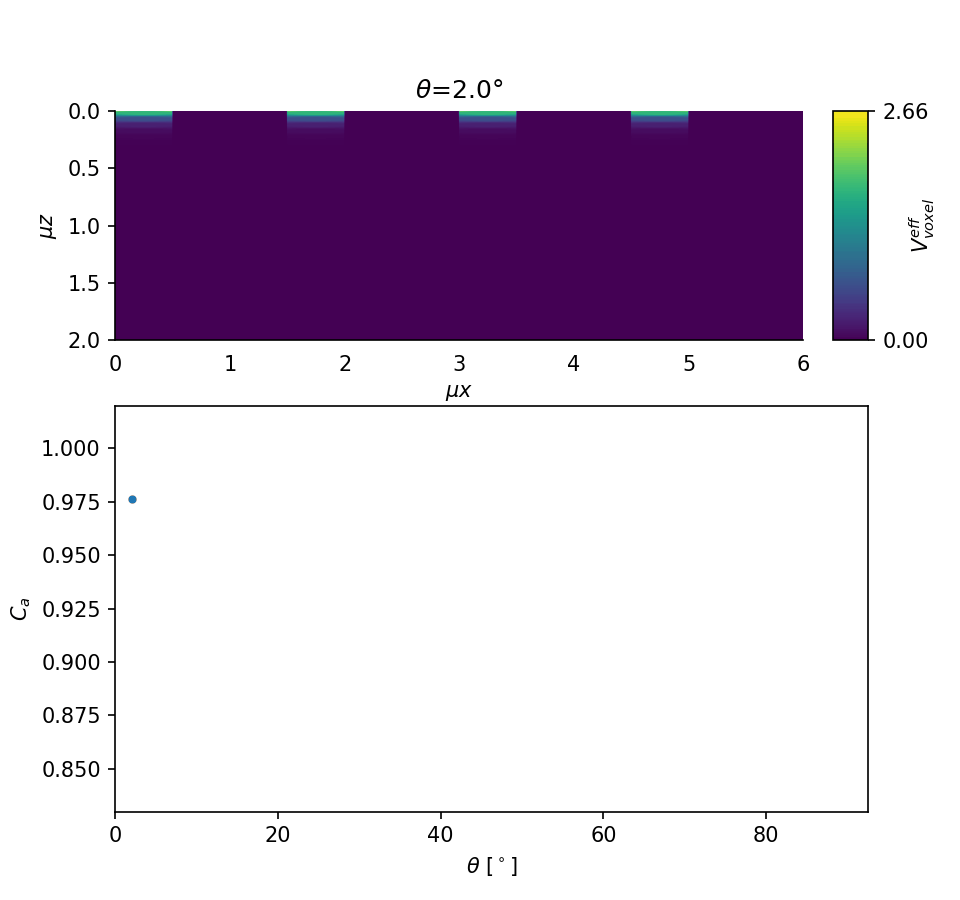

Supplement: Supplementary file 1 [file a-80-00315-sup1.gif]

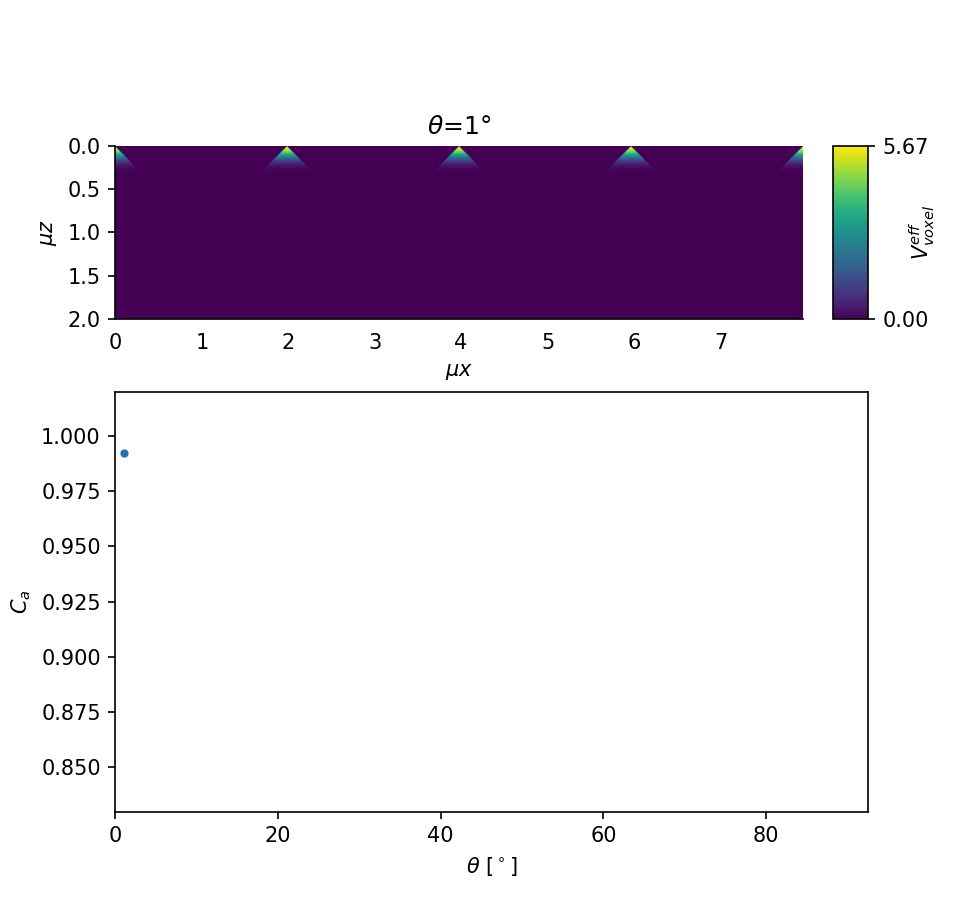

Supplement: Supplementary file 2 [file a-80-00315-sup2.gif]
