# Supplementary material for: Universal simulation of absorption effects for X-ray diffraction in reflection geometry
Source: Acta Crystallogr A Found Adv. 2024 Jun 7;80(Pt 4):315–28. doi: 10.1107/S2053273324003292 (PMC11216610; doi:10.1107/S2053273324003292)
Supplement: Supplementary file 5 [file a-80-00315-sup5.pdf]

FOUNDATIONS
ADVANCES

Volume 80 (2024)

Supporting information for article:

Universal simulation of absorption effects for X-ray diffraction in reflection geometry

Johannes Dallmann, Jonas Graetz and Rainer Hock

Borie's analytical equations

In Fig. 7 a comparison between the simulated absorption corrections (*dots*) of the triangular surface and Borie's analytical solution (*lines*) is made. In his analytical derivation Borie separates the problem in three angular ranges: From 0 to arctan(1/3)≈18.4°, from arctan(1/3) to 45° and from 45° to 90°. The absorption correction is calculated piecewise from three equations. The equation for the angular range from 45° up to 90° matches the curve shown in Fig. 5 of Borie's original publication. The other two solutions for the angular range below 45° do not reproduce the plotted result. The analytical equation for the angular range from arctan(1/3) to 45° was corrected for typing errors. We highlighted the altered terms in red and underlined them in the corrected equation:

$$
\begin{aligned}
R =& \frac{(\sin\theta+\cos\theta)^2}{2\mu a\sin\theta}\left\{1-\exp\left(-\frac{4\mu a\sin\theta}{(\sin\theta+\cos\theta)^2}\right)\right. \\
&+\cot\theta\exp\left(-\frac{4\mu a\sin\theta}{(\sin\theta+\cos\theta)^2}\right) \\
&\quad\times\left[\exp\left(-\frac{4\mu a\sin^2\theta}{\cos\theta(\sin\theta+\cos\theta)^2}\right)-\exp\left(-\frac{4\mu a\cos\theta}{(\sin\theta+\cos\theta)^2}\right)\right] \\
&-\frac{1}{2\cos\theta\sin\theta}\exp\left(-\frac{4\mu a\sin\theta}{(\sin\theta+\cos\theta)^2}\right) \\
&\quad\times\left[1-\exp\left(-\frac{4\mu a\cos\theta}{(\sin\theta+\cos\theta)^2}\right)\right] \\
&+\cot\theta\exp\left(-\frac{4\mu a(2\cos\theta+\sin\theta)}{\cos\theta(\cos\theta+\sin\theta)}\right) \\
&\quad\times\left[1-\exp\left(-\underline{\frac{2\mu a}{\cos\theta}}\right)\right]^{-1}\left[\exp\left(\frac{2\mu a}{\cos\theta}\right)-\exp\left(\frac{\mu a(3\cos\theta-\sin\theta)}{\cos\theta(\sin\theta+\cos\theta)}\right)\right]^2 \\
&-(5\cos\theta-\sin\theta)(\cos\theta-\sin\theta)\cot\theta\exp\left(\underline{-\frac{4\mu a\sin\theta}{\cos\theta(\sin\theta+\cos\theta)}}\right) \\
&\quad\times\left[(3\cos\theta-\sin\theta)^2\left[1-\exp\left(-\frac{2\mu a}{\cos\theta}\right)\right]\right]^{-1} \\
&\quad\left.\times\left[1-\exp\left(-\frac{\mu a(3\cos\theta-\sin\theta)}{\cos\theta(\sin\theta+\cos\theta)}\right)\right]^2\right\}
\end{aligned}
$$

After these corrections, the plot fits very good to the ray-tracing simulations (see Fig. 8). We were unable to correct the equation for the region of smallest angles below 18.4°. While his plot matches very well with our simulations, the equation given does not. From this discrepancy stems the displayed missing congruence between our simulations, Borie's Fig. 5 and the plot of the given equation.

We point out, that Fig. 5 in (Borie, 1981) was already published in (Trucano & Battermann, 1970) and is cited therein as 'After an unpublished calculation of Borie'. Since our simulation confirm the plotted result of Borie we believe there occurred an error in the publication of the equations in (Borie, 1981).
